# Accuracy of automated amygdala MRI segmentation approaches in Huntington's disease in the IMAGE‐HD cohort

**DOI:** 10.1002/hbm.24918

**Published:** 2020-02-07

**Authors:** Bonnie Alexander, Nellie Georgiou‐Karistianis, Richard Beare, Lotta M. Ahveninen, Valentina Lorenzetti, Julie C. Stout, Yifat Glikmann‐Johnston

**Affiliations:** ^1^ Turner Institute for Brain and Mental Health, School of Psychological Sciences Monash University Melbourne Victoria Australia; ^2^ Murdoch Children's Research Institute Melbourne Victoria Australia; ^3^ Department of Medicine Monash University Melbourne Victoria Australia; ^4^ School of Psychology Australian Catholic University Melbourne Victoria Australia

**Keywords:** amygdala, atrophy, Huntington's disease, segmentation, subcortical, tracing

## Abstract

Smaller manually‐segmented amygdala volumes have been associated with poorer motor and cognitive function in Huntington's disease (HD). Manual segmentation is the gold standard in terms of accuracy; however, automated methods may be necessary in large samples. Automated segmentation accuracy has not been determined for the amygdala in HD. We aimed to determine which of three automated approaches would most accurately segment amygdalae in HD: FreeSurfer, FIRST, and ANTS nonlinear registration followed by FIRST segmentation. T1‐weighted images for the IMAGE‐HD cohort including 35 presymptomatic HD (pre‐HD), 36 symptomatic HD (symp‐HD), and 34 healthy controls were segmented using FreeSurfer and FIRST. For the third approach, images were nonlinearly registered to an MNI template using ANTS, then segmented using FIRST. All automated methods overestimated amygdala volumes compared with manual segmentation. Dice overlap scores, indicating segmentation accuracy, were not significantly different between automated approaches. Manually segmented volumes were most statistically differentiable between groups, followed by those segmented by FreeSurfer, then ANTS/FIRST. FIRST‐segmented volumes did not differ between groups. All automated methods produced a bias where volume overestimation was more severe for smaller amygdalae. This bias was subtle for FreeSurfer, but marked for FIRST, and moderate for ANTS/FIRST. Further, FreeSurfer introduced a hemispheric bias not evident with manual segmentation, producing larger right amygdalae by 8%. To assist choice of segmentation approach, we provide sample size estimation graphs based on sample size and other factors. If automated segmentation is employed in samples of the current size, FreeSurfer may effectively distinguish amygdala volume between controls and HD.

## 1 INTRODUCTION

Changes in emotion processing in Huntington's disease (HD) typically manifest in difficulties in recognizing facial expressions, and are part of a range of cognitive, psychiatric, and motor symptoms observed in this disorder (Bates et al., 2015; Henley et al., 2012; Papoutsi, Labuschagne, Tabrizi, & Stout, 2014; Paulsen, Ready, Hamilton, Mega, & Cummings, 2001; Stout et al., 2011). The atrophy seen in HD occurs in a spatiotemporally specific pattern (Fonteijn et al., 2012; Rosas et al., 2008), with some atrophy detectable during the presymptomatic (e.g., Bates et al., 2015; Ross et al., 2014) phase of the disease, that is, well before diagnosable signs and symptoms are present (Aylward et al., 2004; Paulsen, 2010). Neuroimaging studies in HD often focus on characterizing atrophy across stages of the condition, and clarifying relationships between regional atrophy and other symptoms. The amygdala has recently received increased attention in HD research because of its role in emotion processing deficits (e.g., Kipps, Duggins, McCusker, & Calder, 2007; Mason et al., 2015). In the amygdala, volumetric MRI studies are critical in clarifying how atrophy relates to deficits in emotion processing, although changes are subtle, relative to the marked atrophy seen in the striatum (Aylward et al., 1994, 2004; Domínguez et al., 2013; Domínguez et al., 2016; Fonteijn et al., 2012; Papoutsi et al., 2014; Poudel et al., 2014; Tabrizi et al., 2009; Vonsattel et al., 1985). Even in healthy individuals, the amygdala is somewhat challenging to segment on MRI due to its heterogeneous intensity, and tissue boundaries that can appear indistinct (Chupin et al., 2009; Entis, Doerga, Barrett, & Dickerson, 2012). In terms of MRI segmentation accuracy in general, manual tracing is regarded the “gold standard”; however, manual segmentation is most often prohibitively time consuming and in the context of large cohort MRI studies (which are common in HD research) is rarely feasible (e.g., Hammers et al., 2003; Heckemann, Hajnal, Aljabar, Rueckert, & Hammers, 2006). Therefore, automatic methods for segmentation are essential.

FreeSurfer (Fischl et al., 2002) and FIRST (Patenaude, Smith, Kennedy, & Jenkinson, 2007; Patenaude, Smith, Kennedy, & Jenkinson, 2011) are two commonly used, freely available segmentation tools that label subcortical structures and output regional volumes. Amygdala‐specific segmentation tools have also been developed (Collins & Pruessner, 2010; Hanson et al., 2012; Saygin et al., 2017) though some are not publicly available (Collins & Pruessner, 2010; Hanson et al., 2012). In HD, many volumetric studies have used FreeSurfer or FIRST, which label multiple parcellated regions throughout the brain. Thus, we have focused on these widely used tools. The accuracy of FreeSurfer and FIRST has been previously compared with reference to gold standard manual segmentation in normal and clinical populations, and in different subcortical brain regions (Doring et al., 2011; Merkel et al., 2015; Morey et al., 2009; Mulder et al., 2014; Pardoe, Pell, Abbott, & Jackson, 2009; Perlaki et al., 2017; Schoemaker et al., 2016). Results have been mixed, and vary based on sample and brain region. With regards to the amygdala specifically, Morey et al. (2009) found that FreeSurfer performed better on some measures of accuracy in healthy adults and in a small sample (*n* = 9) of individuals with major depressive disorder. Schoemaker et al. (2016) found mixed results in preadolescent children, and suggested that segmentations derived via both FreeSurfer and FIRST may require manual corrections. These results, however, are not generalizable to HD, which has a unique neuropathological basis. The atrophy in amygdala and surrounding structures that occurs during the course of HD (Ahveninen, Stout, Georgiou‐Karistianis, Lorenzetti, & Glikmann‐Johnston, 2018), may influence the accuracy of amygdala segmentation. It is thus imperative to determine which of these pipelines is most appropriate for this clinical cohort.

Both FreeSurfer and FIRST pipelines implement registration and segmentation routines, and utilize Bayesian approaches to fit models that draw upon manually labeled training sets. There are many aspects of the processing pipelines that differ between the two tools, including the type of model used. Another point of difference is the registration approach used, and we focused on this aspect in the current article. FreeSurfer's subcortical pipeline performs initial affine registration to the MNI 305 template (Evans, 1992), initial labeling, bias correction, then nonlinear registration to the MNI 305 template, which deforms the target image so it can match the template as closely as possible (Fischl et al., 2002, 2004). FreeSurfer uses a model that incorporates anisotropic nonstationary Markov Random Fields to fit labels based on intensity as well as spatial location relative to neighboring structures. In comparison, FIRST performs an affine‐only registration to the MNI152 nonlinear 1 mm template (Fonov et al., 2011) using FLIRT, and transforms the model to native space in order to capitalize on intensity information in the noninterpolated image. The model employed in FIRST is a Bayesian Appearance Model, which fits deformable shape meshes based on conditional probability of shape and intensity information (Patenaude et al., 2011). FIRST's use of linear transformations rather than nonlinear warping restricts how closely structures in a training set can be mapped onto those in a target image during the registration step. However, this is overcome by the Bayesian framework allowing shape meshes to deform beyond the shapes existing in the training set in order to match the target more closely (Patenaude et al., 2011). Considering the abnormal amygdala size seen in HD (Ahveninen et al., 2018), we were interested to determine whether segmentations performed by FIRST may be improved by performing initial nonlinear warping of the data to template space.

In the current article, we utilized the Australian‐based IMAGE‐HD cohort (including 35 pre‐HD, 36 symp‐HD, and 35 healthy controls), for which manual amygdala segmentation had been performed by Ahveninen et al. (2018). We aimed to identify the accuracy with which three automated segmentation approaches would segment the amygdala for this sample, by comparing the output of each pipeline with the manual segmentation, thereby identifying which is most appropriate for use in HD. We also aimed to provide estimates of sample sizes required to produce amygdala volumes that are statistically differentiable between HD and controls, and between pre‐HD and symp‐HD, for each automated approach. The automated approaches tested were FreeSurfer, the complete default FIRST pipeline, and FIRST's segmentation algorithm applied to whole‐head images bias corrected and nonlinearly transformed into MNI space using ANTS.

## 2 METHODS

### 2.1 Participants

The sample comprised 106 participants aged 23 to 72 years from the IMAGE‐HD study (Domínguez et al., 2013, 2016; Georgiou‐Karistianis et al., 2013). These included 34 healthy controls, 35 presymptomatic huntingtin gene expansion carriers who had not developed motor symptoms at the time of scanning (termed ‘pre‐HD’), and 36 individuals with early stage symptomatic HD (‘symp‐HD’). One control participant was excluded due to failed MRI labeling via FIRST (described further in Section 2.3.2), resulting in 34 controls. HD participants were genetically confirmed to have the huntingtin gene expansion (≥38 CAG repeats), and were between 23 and 70 years of age, with no history of major neurological illness (except HD), significant head injury, or non‐HD‐related psychiatric disturbances. Participants with a UHDRS total motor score (TMS) ≤ 5 were included in the pre‐HD group, and those with a UHDRS TMS score of 5 or above were included in the symp‐HD group (Domínguez et al., 2013). Participants with pre‐HD had Unified Huntington's Disease Rating Scale (UHDRS) diagnostic confidence levels of less than four, indicating that they had not received the HD diagnosis (Huntington Study Group, 1996). Participants with symp‐HD had Stage 1 or Stage 2 HD.

Groups significantly differed in terms of age (*F*[2,102] = 8.701, *p* < .001), with the symp‐HD group being older than the pre‐HD group (*p* = .001), as is typically observed given the progressive nature of HD. The symp‐HD group was also older than the control group (*p* = .003). We chose to retain all participants rather than using subsets of closer age in order to account for the progressive brain atrophy that is a fundamental characteristic of HD, and becomes more severe with older age. In doing so, we accept that there will be some proportion of atrophy in the symp‐HD group attributable to normal ageing. Pre‐HD and control groups did not differ in terms of age (*p* = .890). See Table 1 for participants' demographic information and clinical data.

**Table 1:** Demographic information for participants in HD groups (reproduced from Ahveninen et al., 2018), and for the subset of controls with successful segmentation for all methods

	Presymptomatic HD	Symptomatic HD	Controls (with successful segmentation)
*n*	35	36	34
Males (%)	14 (40%)	21 (58%)	12 (35%)
Females (%)	21 (60%)	15 (42%)	22 (65%)
Age (mean ± *SD*)	41.59 ± 10.00	51.91 ± 9.36	42.58 ± 13.78
(range)	(23.93–65.29)	(38.99–70.84)	(24.38–72.98)
UHDRS TMS (mean ± *SD*)	.94 ± 1.24	19.47 ± 12.42	‐
(range)	(0–4)	(6–60)	‐
CAG repeats (mean ± *SD*)	42.31 ± 1.97	43.17 ± 2.48	‐
(range)	(39–46)	(40–50)	‐
DBS (mean ± *SD*)	269.70 ± 53.41	379.70 ± 70.02	‐
(range)	(131.64–369.60)	(258.14–556.97)	‐

Abbreviations: DBS = Disease burden score; UHDRS TMS = Unified Huntington's disease rating scale total motor score.

### 2.2 MRI acquisition

MRI scans were acquired on a Siemens Magnetom Trio Tim scanner with a 32‐channel head coil, at Murdoch Children's Research Institute, Royal Children's Hospital, Victoria, Australia. T1‐weighted images were acquired with: 192 slices, slice thickness 0.9 mm, in‐plane resolution 0.8 × 0.8 mm, 320 × 320 field of view, TR = 1,900 ms, TE = 2.59 ms, inversion time 900 ms, flip angle 9°.

### 2.3 Segmentation

#### 2.3.1 Manual segmentation

Manual tracing of amygdala was performed by L.A. using Analyze 12.0 (AnalyzeDirect, Overland Park, KS) according to an existing protocol (Velakoulis et al., 2006) while blind to which group (i.e., control, pre‐HD, or symp‐HD) participants belonged to. Intraclass correlation coefficients (ICCs) indicating intrarater reliability were .89 (right) and .84 (left). ICCs indicating inter‐rater reliability (with Y.G.‐J.) were .88 (right) and .80 (left) (Ahveninen et al., 2018).

#### 2.3.2 Automatic segmentation

##### FreeSurfer

T1‐weighted images were input into the default pipeline of FreeSurfer 6.0, using the ‘recon‐all’ command. The amygdalae were isolated from the resulting ‘aseg’ image.

##### FIRST

FIRST (Patenaude et al., 2011) was run using the 'run_first_all' script, which implements automatic registration to the MNI nonlinear 1 mm template (Fonov et al., 2011), and segmentation. We considered segmentation to have failed for one participant in the control group, due to poor registration resulting in the amygdala label being placed too dorsally, partially overlying basal ganglia structures. Rerunning registration using the ‘first_flirt’ command with different parameters did not improve the registration. Thus, we excluded segmentation for this subject from the dataset. The 34 control individuals listed as participants are those for whom FIRST segmentation was completed successfully, from an initial group of 35 controls.

##### ANTS/FIRST

T1‐weighted images were bias corrected using the ‘N4BiasFieldCorrection’ (Tustison et al., 2010) script in ANTS (Avants et al., 2011). Images were then registered to the MNI 1 mm nonlinear template using ANTS, with the ‘AntsRegistration’ and ‘AntsApplyTransforms’ scripts. An affine transformation was first performed, followed by a nonlinear transformation using symmetric diffeomorphic normalisation (SyN) with cross‐correlation as the similarity metric. Segmentation was then run on the bias corrected, nonlinearly registered T1 images in MNI space using FIRST's ‘run_first’ script, using an identity matrix as the input transformation matrix. FIRST's pipeline also includes bias correction, and we acknowledge that the images thus underwent bias correction multiple times for this method. Resulting segmentations were then transformed from MNI space back to native space with the ‘AntsApplyTransforms’ script, using the inverse of the affine transformation matrix and nonlinear warp image generated by ANTS during the registration of T1 images to the MNI template.

### 2.4 Statistical analysis

We evaluated the accuracy of the automated amygdala segmentation approaches by: (a) computing Dice overlap scores between automated and manual segmentations as a measure of automated segmentation accuracy; (b) determining whether amygdala volume differences between groups detected for manually segmented amygdala, could be detected using automated methods; (c) producing Bland–Altman plots to indicate estimation bias based on amygdala size; (d) comparing volumes of left and right amygdala to indicate potential hemispheric bias in volumes produced by automated methods; and (e) producing sample size estimation graphs, to provide an indication of relative sample sizes required to detect group differences in amygdala volume for each method. Statistical analyses were performed using R (R Core Team, 2018).

#### 2.4.1 Deviations from normality

All amygdala volumes were compared in native space. We tested amygdala volumes and Dice scores for normality, skew, and kurtosis, and found that roughly one‐sixth of the data were not normally distributed. Outliers were also present. We did not transform the data or correct outliers because we wanted to depict the observed distributions of volumes provided by each method as accurately as possible. Due to these violations of normality, we employed nonparametric statistics in all statistical comparisons.

#### 2.4.2 Dice overlap scores

Dice scores (Dice, 1945) are used to indicate the accuracy of segmentation with reference to a “true” segmentation—in this case, manual segmentation, by measuring the proportion of overlap between segmentations. Dice scores range between 0 (no overlap) and 1 (complete overlap). We obtained Dice scores using the ‘overlap’ function in Convert3D (http://www.itksnap.org/c3d/).

#### 2.4.3 Statistical tests of differences in volumes and Dice scores between and within groups

Differences between pairs of measurements within subjects, such as comparisons of left and right hemisphere volumes, were tested using Wilcoxon signed‐rank tests (using the ‘wilcox.test’ function in R). We calculated standardized effect sizes (denoted by *r*) for within subject differences using *r =* z/sqrt(number of observations; Field, 2013). Differences in amygdala volumes between groups were investigated for each segmentation method using Wilcoxon rank sum tests. The median of the volume difference between a sample from each group, in mm^3^, was reported for these tests. *P*‐values for each of these sets of tests were both False Discovery Rate corrected (FDR; Benjamini & Hochberg, 1995) and Bonferroni corrected to control for multiple comparisons. We report the uncorrected, FDR corrected, and Bonferroni corrected values. To investigate differences in Dice scores across the three automated segmentation approaches, we used Kruskall–Wallis tests (‘kruskal.test’ in R), which are a nonparametric equivalent to an ANOVA, performed on rank data.

#### 2.4.4 Plots of amygdala volumes

We generated scatterplots of volumes between segmentation techniques. Intraclass correlation coefficients (the quantitative measure of correlation relevant for repeated measures comparisons) could not be computed due to the violations of normality in some subsets of the data, and thus are not reported.

Bland–Altman plots (Bland & Altman, 1986), which indicate the difference in estimation between two methods (i.e., between manual and automated segmentation here), were generated to provide an indication of possible bias in volume estimation. Similarly to the approach of Schoemaker et al. (2016), we used the manually segmented amygdala volumes on the *X*‐axis (see also Krouwer, 2008, for justification of this method), and included regression lines to assist with interpretation.

#### 2.4.5 Sample size estimation

Sample size estimation was performed using the R package ‘pwr’ and was based on two‐tailed, two‐sample *t*‐tests. We computed estimates for the size per group of the sample for a range of effect sizes, expressed as amygdala volume difference in mm^3^, for each segmentation type. The sample size estimates were computed separately based on two separate sets of values. First, they were computed based on observed power and *p*‐values obtained for *t*‐tests performed on the amygdala volumes for manually segmented data. This allowed us to establish the sample size that would be required for each automated method to produce the equivalent capacity to differentiate between groups as seen for manual segmentation. Second, to determine minimum sample sizes required to differentiate between each group under conditions of high power, estimates were computed using power = 0.8 and *p* = .05 (as per Morey et al. (2009)). Sample size estimates were computed for left and right amygdala volumes separately because *t*‐tests, upon which the estimates were based, must be performed separately for each hemisphere.

## 3 RESULTS

### 3.1 Amygdala volumes and volume differences between segmentation methods

**Figure 1:**
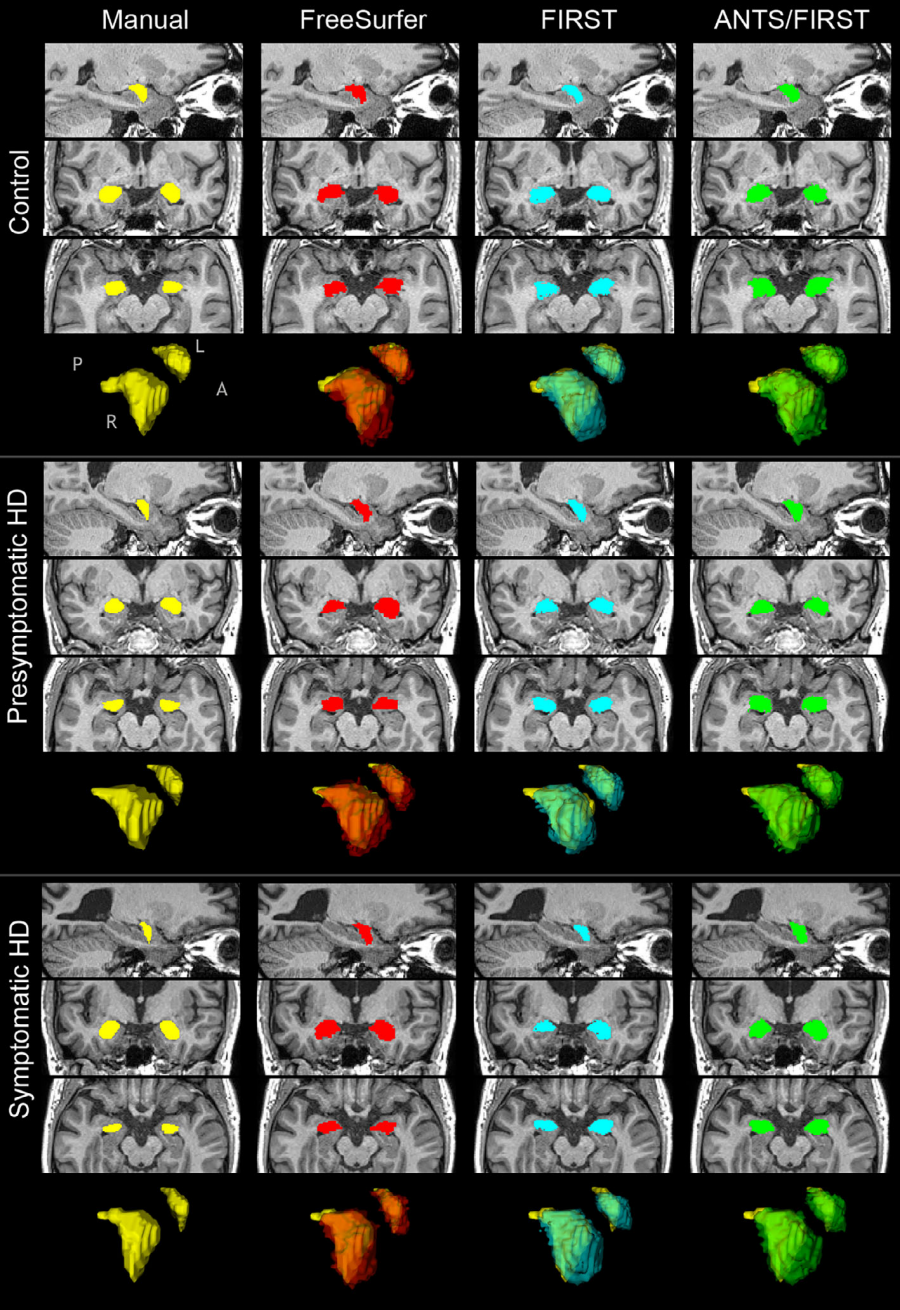
Examples of segmentations resulting from each technique, for a single participant from each group. Sagittal, coronal, and axial slices are displayed for each combination of participant and segmentation technique, shown in neurological orientation (left is left). Surface mesh representations of segmented amygdala based on each technique are displayed for the same individual participants. Yellow meshes are manual segmentations. Red, blue, and green meshes are for automated techniques, and are overlaid on the manually segmented meshes to illustrate differences in morphology. Anatomical axis descriptors: ‘A’ = anterior, ‘L’ = left, ‘P’ = posterior, ‘R’ = right

**Figure 2:**
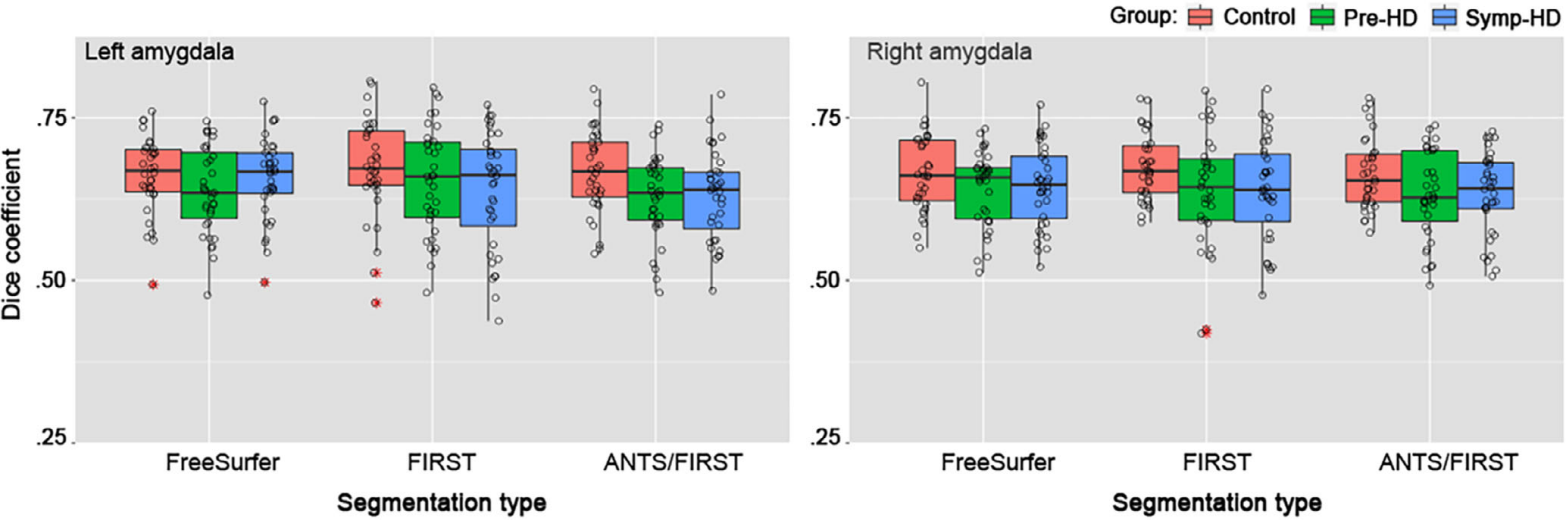
Dice coefficients indicating overlap between automatic amygdala segmentations (FreeSurfer, FIRST, ANTS/FIRST) and the gold standard manual tracing. Shown for controls (red plots), pre‐HD (green plots), and symp‐HD (blue plots). Left panel: left hemisphere. Right panel: right hemisphere. Boxplot canter hinge indicates median, and top and bottom indicate 25th and 75th percentiles. Whiskers extend to the furthest value within ±1.5 × the interquartile range. Outliers (outside of ±1.5 × the interquartile range) are indicated by asterisks. ‘Con’ = control, ‘pre’ = pre‐HD, ‘sym’ = symp‐HD

All automated methods overestimated amygdala volumes when compared to manually segmented volumes. Visual inspection indicated that amygdala segmentations extended further anteriorly for all automated methods compared with manual segmentations (Figure 1). This difference was most marked for ANTS/FIRST, followed by FreeSurfer, then FIRST (Figure 2 and Table 2). Density plots of amygdala volumes are provided in Figure S1.

**Table 2:** Mean amygdala volume (*SD* in parentheses) for each subset of group, hemisphere, and segmentation method, and differences between automated and manual volumes

Hem.	Group	Manual seg.	FreeSurfer		FIRST		ANTS/FIRST	
		Volume (*SD*)	Volume (*SD*)	% diff. (*SD*)	Volume (*SD*)	% diff. (*SD*)	Volume (*SD*)	% diff. (*SD*)
Left	All	984.02 (203.16)	1,588.25 (254.28)	61.40 (20.06)	1,430.21 (260.79)	45.34 (28.85)	1,774.77 (224.83)	80.36 (21.66)
Left	Con	1,142.17 (212.02)	1,719.59 (209.08)	50.55 (15.76)	1,465.83 (268.49)	28.34 (27.48)	1836.99 (181.13)	60.83 (16.71)
Left	Pre	945.62 (152.52)	1,569.17 (214.54)	65.94 (17.54)	1,422.04 (240.88)	50.38 (23.80)	1,788.24 (229.15)	89.11 (18.90)
Left	Symp	867.61 (130.88)	1,479.13 (277.38)	70.48 (27.64)	1,403.54 (274.91)	61.77 (31.07)	1,701.17 (243.20)	96.08 (27.29)
Right	All	986.59 (199.50)	1,707.77 (246.57)	73.10 (19.20)	1,467.27 (278.96)	48.72 (30.04)	1,766.40 (240.82)	79.04 (21.67)
Right	Con	1,128.22 (213.90)	1815.97 (249.18)	60.96 (16.88)	1,437.67 (287.94)	27.43 (23.78)	1807.55 (240.58)	60.21 (15.68)
Right	Pre	955.76 (149.16)	1,675.69 (201.59)	75.33 (17.72)	1,469.82 (272.92)	53.79 (29.29)	1,796.92 (266.85)	88.01 (27.19)
Right	Symp	878.86 (142.80)	1,633.75 (253.41)	85.90 (23.41)	1,493.58 (281.00)	69.95 (29.81)	1,696.72 (202.41)	93.06 (18.26)

Abbreviations: ‘% Diff’ = percentage difference in amygdala volumes between manual segmentations and automated segmentations (*SD* in parentheses). ‘All’ = data for all groups combined; ‘Con’ = controls; ‘Hem’ = hemisphere; ‘Pre’ = presymptomatic HD; ‘Symp’ = symptomatic HD.

### 3.2 Dice coefficients based on segmentation type

We sought to examine the extent of overlap between amygdala segmentations produced manually and those produced automatically, by calculating Dice scores (Dice, 1945).

Average Dice scores (across all groups and both hemispheres) were 0.65 for FIRST, 0.64 for ANTS/FIRST, and 0.61 for FreeSurfer. Dice coefficients for each group and hemisphere are shown in Figure 2. There was no significant difference in Dice scores across the three automated segmentation approaches, for left amygdala (*χ* ^2^ = 3.703, df = 2, *p* = .157), or right amygdala (*χ* ^2^ = 1.591, df = 2, *p* = .451). Similarly, we found no significant differences in Dice scores for data broken down into groups: controls (left: *χ* ^2^ = 0.834, df = 2, *p* = .659, right: *χ* ^2^ = 0.912, df = 2, *p* = .634), pre‐HD (left: *χ* ^2^ = 1.725, df = 2, *p* = .422, right: *χ* ^2^ = 0.601, df = 2, *p* = .741), and symp‐HD (left: *χ* ^2^ = 2.931, df = 2, *p* = .231, right: *χ* ^2^ = 0.579, df = 2, *p* = .749).

### 3.3 Differences in amygdala volume between groups detected for volumes derived via each segmentation method

**Figure 3:**
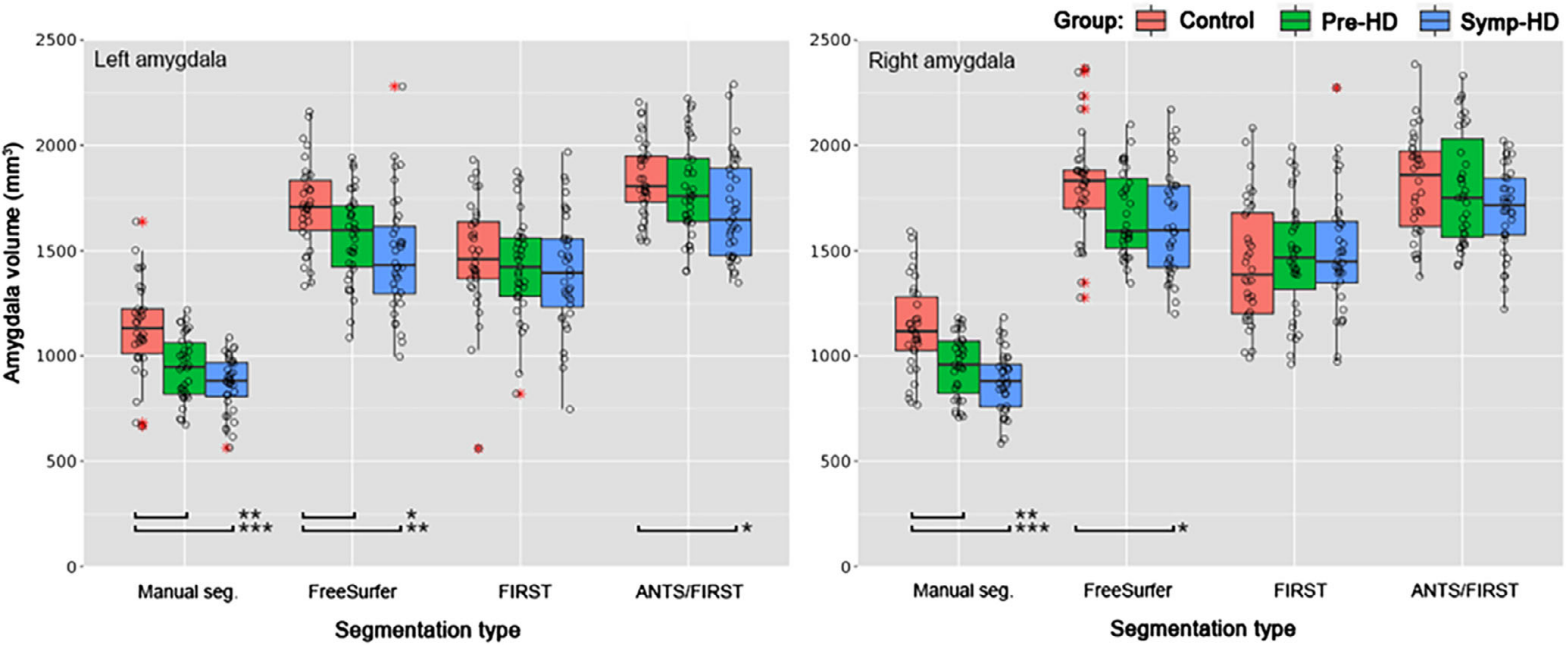
Amygdala volumes in mm^3^ based on segmentation type in controls (red plots), pre‐HD (green plots), and symp‐HD (blue plots). Left panel: left amygdala. Right panel: right amygdala. Boxplot center hinge indicates median, and top and bottom indicate 25th and 75th percentiles. Whiskers extend to the furthest value within ±1.5 × the interquartile range. Outliers (outside of ±1.5 × the interquartile range) are indicated by red asterisks. ***Wilcoxon rank sum test indicated a significant difference in volume between groups with *p* < .05 (FDR corrected), ** *p* < .01, *** *p* < .001

Next, we examined the extent to which group differences in amygdala volumes (controls vs. pre‐HD, controls vs. symp‐HD, and pre‐HD vs. symp‐HD) were found for different segmentation methods, using Wilcoxon rank sum tests (FDR‐corrected for multiple comparisons). Manual segmentation provided volumes that allowed groups to be most successfully differentiated. Significant differences in manually segmented volumes were found between controls and pre‐HD, and controls and symp‐HD, for both the right and left amygdala. Manually segmented volumes were differentiable between the two HD groups only when uncorrected for multiple comparisons (*p* = .030), but not with FDR correction (*p* = .078). For FreeSurfer segmentation, differences in amygdala volumes were not detected between pre‐HD and symp‐HD groups in either hemisphere, though differences were found between controls and each HD group in the left amygdala, and between controls and symp‐HD in the right amygdala. No significant group differences in volumes were found for segmentations derived via FIRST. For ANTS/FIRST, the only volume difference detected was between controls and symp‐HD in the left amygdala. Results for these comparisons are illustrated Figure 3, and complete statistics including uncorrected, FDR‐corrected, and Bonferroni‐corrected *p*‐values are provided in Table S1.

### 3.4 Associations between manually and automatically segmented volumes, and assessment of estimation bias

**Figure 4:**
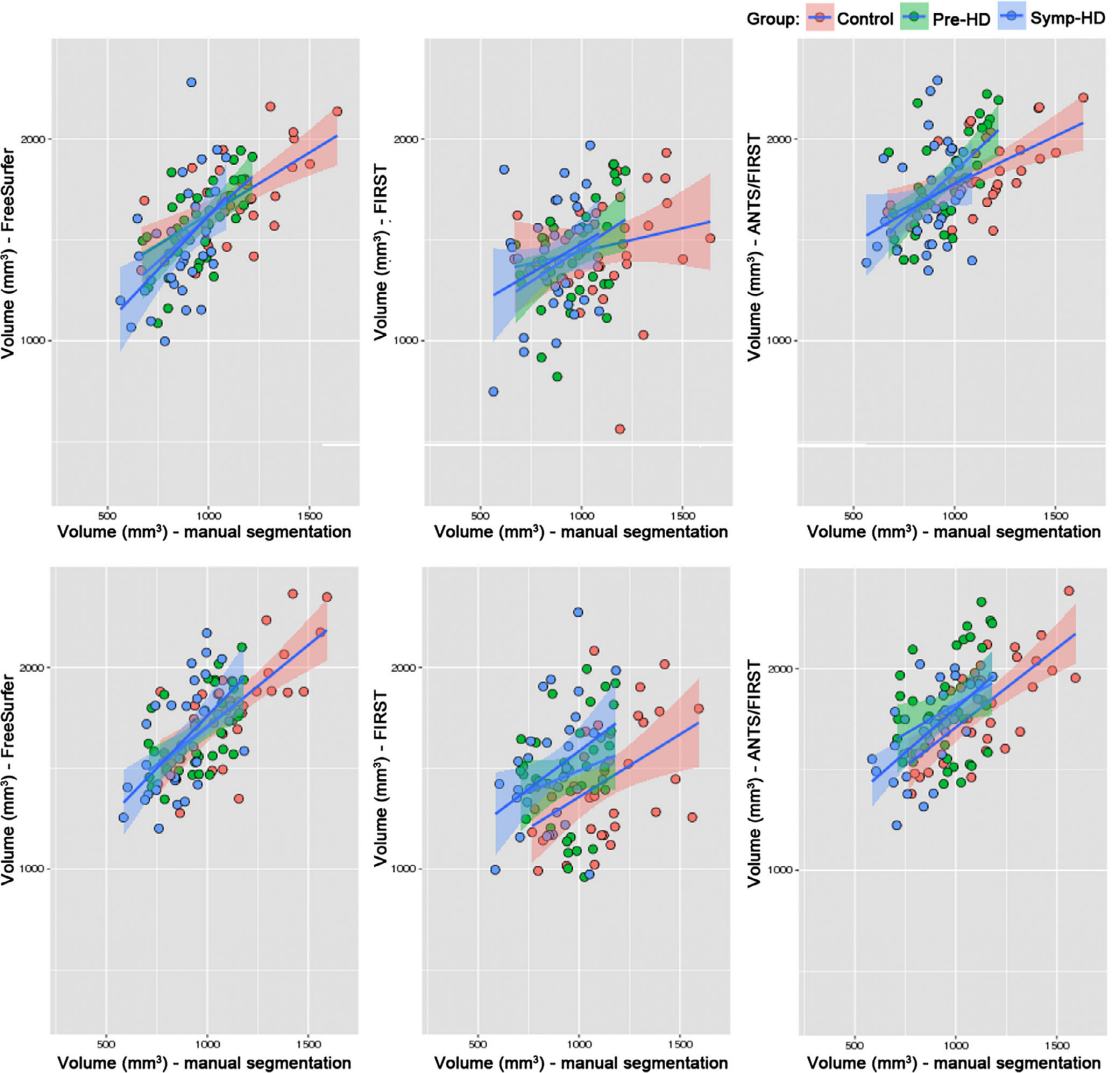
Scatterplots of automated versus manual amygdala volume in mm^3^ for each segmentation approach, with regression lines based on linear models for controls (red plots), pre‐HD (green plots) and symp‐HD (blue plots). Shaded areas are 95% confidence intervals. Top row: left amygdala. Bottom row: right amygdala

**Figure 5:**
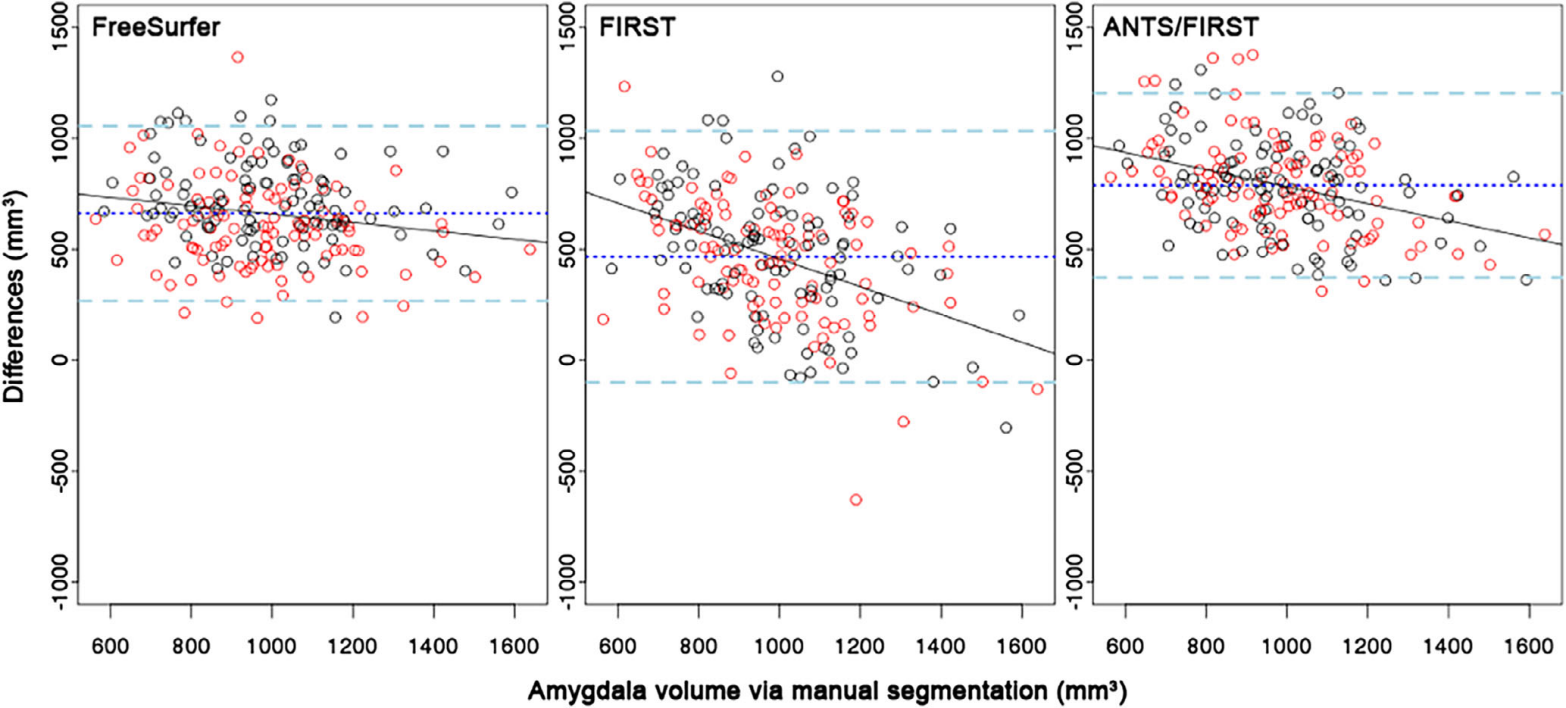
Bland–Altman plots of amygdala volume for each automated segmentation technique, versus manual segmentation, for all groups combined. *X*‐axis: manually segmented amygdala volume. *Y*‐axis: difference in amygdala volume between manual segmentation and the automated segmentation. Red: left amygdala. Black: right amygdala. Dark blue line: mean difference. Light blue lines: upper and lower limits of differences, that is, mean difference ± 1.96 × *SD*. Black line: regression line from linear model

Intraclass correlation coefficients (i.e., the appropriate measure of correlation for measurements within subjects), could not be computed because the data were nonparametric. We therefore plotted the data to illustrate the associations between automatically and manually segmented amygdala volumes for each segmentation method (Figure 4). From visual inspection, these associations appear strongest for FreeSurfer, then ANTS/FIRST, and weakest for FIRST. For FIRST, the regression line between automated and manually segmented volumes appears to be different between groups, particularly in the right hemisphere where the intercept of the regression line for controls appeared lower than that for symp‐HD. The relationship between manually and automatically segmented volumes for each automated method was explored further using Bland–Altman plots (Figure 5), which are used to indicate estimation bias.

The negative slopes of the regression lines in each panel indicate that all automated segmentation approaches produced an estimation bias: overestimation of volumes was more severe for smaller amygdalae, and less severe for larger amygdalae. This bias was relatively small for FreeSurfer, though quite marked for FIRST, and somewhat reduced for ANTS/FIRST compared to that for FIRST.

### 3.5 Right versus left amygdala volume comparisons within segmentation techniques

We compared left amygdala volumes with right volumes within each segmentation type for all data, and then for each HD group. Wilcoxon signed rank tests indicated amygdala volumes segmented using FreeSurfer were statistically significantly larger in the right hemisphere than the left hemisphere, for all groups combined (*p* < .001, FDR corrected) and for each group separately (all *p* < .01). Complete statistics for these comparisons are listed in Table S2. Right amygdala volumes were on average 7.6% larger than left (5.5% for controls, 6.4% for pre‐HD, and 9.4% for symp‐HD). No hemispheric volume differences were found for FIRST, ANTS/FIRST, or manual segmentations.

### 3.6 Sample size estimation

**Figure 6:**
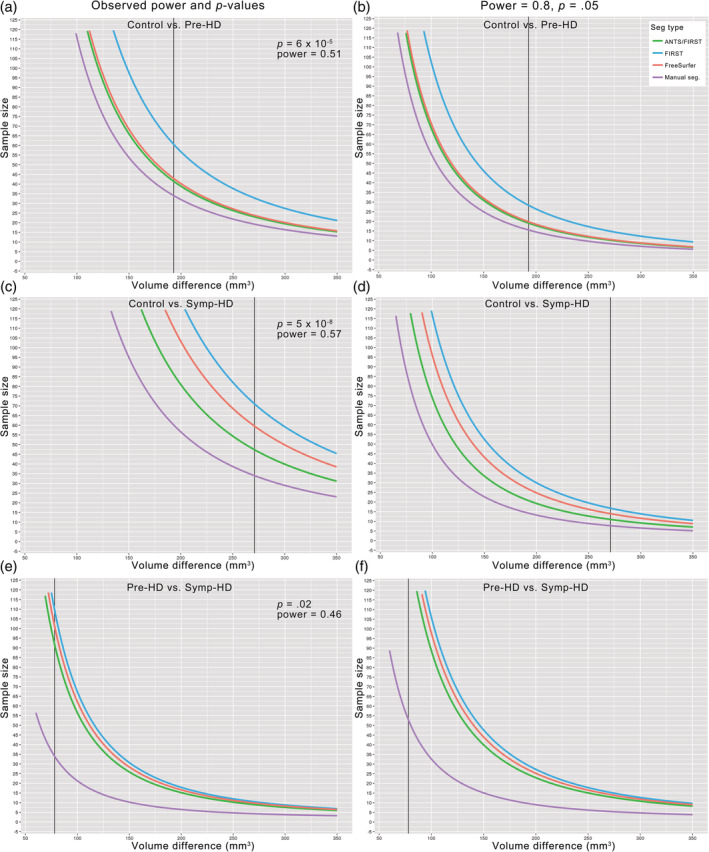
Sample size estimation for ability to statistically detect differences in amygdala volumes between groups. Based on observed power and observed *p*‐values (left), and power = 0.8 and *p* = .05 (right) for comparisons between control and pre‐HD (a,b), control and symp‐HD (c,d), and pre‐HD and symp‐HD (e,f). Estimates assume parametric data, and are based on *t*‐tests performed on left amygdala volumes (corresponding graphs based on tests performed on right amygdala volumes are presented in Figure S2). Vertical black lines indicate observed mean amygdala volume difference between groups in mm^3^. The *Y*‐axis limits are sample sizes comprising between *n* = 0 and *n* = 125 per group. This upper limit was chosen for clarity of visualization

Sample size estimates for detection of amygdala volumes between groups, for a range of effect sizes (indicated by difference in amygdala volume between groups in mm^3^), are shown in Figure 6. Graphs in Figure 6 are based on statistics for left hemisphere amygdala volumes, as described in Section 2.4.5. Graphs based on statistical comparisons of right hemisphere volumes are provided in Figure S2.

Sample size estimation indicated that for all comparisons, the sample size required to detect amygdala volume differences between groups was smallest for manual segmentation, and largest for FIRST. For example, in section (e) of Figure 6, the difference observed between manually segmented amygdala volumes in pre‐HD and symp‐HD with the current sample size (*n* ~ 34 per group) is seen at the point where the purple line crosses the black vertical line. Following the black line upward from this point illustrates that to detect the same effect with automated approaches requires a larger sample size of approximately *n* = 92 per group for ANTS/FIRST, *n* = 102 for FreeSurfer, and *n* = 110 for FIRST. Figure 6 part f) illustrates the sample sizes required to detect this effect with *p* = .05 given high power (0.8) with approximately *n* = 53 per group for manual segmentation, and substantially higher for automated methods, such that the gradient of the curves began to steepen rapidly around this point, producing values higher than *n* = 125.

## 4 DISCUSSION

For the first time, we examined the accuracy with which different automated segmentation methods segmented the amygdala at different stages of HD. The statistical power in our study, provided by the large sample size and equally‐sized subgroups of the IMAGE‐HD cohort, facilitated the detection of amygdala volume differences between groups in manually segmented data, which in turn provided a reference for the assessment of accuracy of automated methods. Thus this study provides information that is useful for guiding methodological choices in volumetric studies in HD, and may help inform interpretation of existing amygdala volumetric results.

We found that all automated methods overestimated amygdala volume for all groups, compared with volumes produced with manual segmentation. Specifically, FreeSurfer overestimated volumes by 61% on average, FIRST overestimated volumes by 45% on average, and ANTS/FIRST by 83% on average. These inflated volume estimates would be problematic in any context in which absolute amygdala volume is of primary importance. They also illustrate that amygdalae that have been segmented with different methods cannot be directly compared in terms of absolute volume, such as where segmentation methods differ across studies.

Qualitatively, amygdala segmentations extended further anteriorly for all automated methods compared with manual segmentations. A factor contributing to this may be that the amygdala segmentation protocols differ between manual and automated segmentation techniques. The manual segmentations performed by Ahveninen et al. (2018) and used here were delineated according to the widely‐used protocol specified in Velakoulis et al. (2006). The training sets for both FreeSurfer and FIRST employ manually segmented images provided via the Center for Morphometric Analysis (see https://fsl.fmrib.ox.ac.uk/fsl/fslwiki/FIRST, and http://freesurfer.net/fswiki/SubcorticalSegmentation, wherein it is indicated that original segmentation protocols are described in Filipek, Richelme, Kennedy, and Caviness (1994)). Velakoulis et al. specify the anterior boundary of the amygdala as, “the section anterior to the appearance of the optic chiasm” (p. 142). In comparison, Filipek et al. do not explicitly specify an anterior boundary. It is possible that the amygdala labels in the training sets for FreeSurfer and FIRST extend more anteriorly than the boundary specified by Velakoulis and colleagues, resulting in amygdala segmentations of the current data being more extensive anteriorly. However, this cannot be confirmed on the basis of the provided protocols (see Supporting Information for complete protocol descriptions).

A potential caveat relating to manual segmentation in HD is that atrophy in the amygdala, surrounding or widespread regions may be visibly noted on the scans. This may compromise the blinding of those performing manual segmentation to participants' group membership, which could potentially lead to a systematic bias in amygdala volume between groups. Although this possibility cannot be eliminated for the current data, the tracing protocol provides clear anatomical boundaries, and inter‐rater reliability was found to be high, so we are reasonably confident in the accuracy of the segmentations in the presence of atrophy.

Quantitatively, we indicated accuracy of automated segmentations by calculating Dice scores, which represent the proportion of overlap between label images produced by manual and automated segmentation. Dice scores ranged between 0.6 and 0.65, and did not statistically differ between automated segmentation approaches. These unimpressive scores are not surprising considering that: (a) the amygdala is a challenging structure to segment, so high automated labeling accuracy would not be expected, and; (b) the automated methods greatly overestimated amygdala volume, thus the proportion of overlap between a given automated (large) label, and the corresponding manual (small) label, would be small because much of the automated label extends outside of the manual label. Accordingly, the more extensive volume overestimation produced by FreeSurfer and ANTS/FIRST, compared to that for FIRST, may have also reduced the average Dice scores for these approaches compared to that for FIRST. Since no automated technique produced segmentations that markedly altered Dice scores, this metric may not be the most useful indicator of segmentation accuracy for this data.

In the context of clinical studies, the ability to accurately detect existing volume differences between HD groups and controls, and between pre‐ and symp‐HD, may be the most useful criterion for assessing which segmentation approach to employ. Here, manual segmentation produced volumes that were most easily differentiated between groups, with controls readily differentiable from both symp‐HD and pre‐HD in both the left and right amygdala. Manual segmentation also produced right amygdala volumes that were statistically different between pre‐HD and symp‐HD when uncorrected for multiple comparisons, but not with FDR correction. Other methods did not differentiate amygdala volume in pre‐HD and symp‐HD. Therefore, in studies where this distinction is important, manual segmentation and a slightly larger sample size may be necessary. Furthermore, in order to more closely characterize where in the amygdala volume differences occurred between groups or time points, the use of shape analysis may be beneficial. FreeSurfer was second most effective at differentiating amygdala volumes between groups, producing segmentations that could distinguish controls from either of the HD groups in left amygdala, and could distinguish controls from symp‐HD in the right amygdala. Where manual segmentation is not feasible, our findings indicate that FreeSurfer is the next most effective method at producing amygdala volumes that preserve differences between groups. FIRST produced volumes that were not statistically different between groups, so we do not recommend using FIRST for segmentation of amygdala in HD in samples of the current size. Incorporating ANTS nonlinear registration with FIRST segmentation only slightly improved the ability to detect differences in amygdala volumes between groups, resulting in a volume difference between controls and symp‐HD only in left amygdala.

The unfavorable results for FIRST may be relevant for the interpretation of previous amygdala volumetric studies in HD that used FIRST. For example, Coppen, Jacobs, van den Berg‐Huysmans, van der Grond, and Roos (2018) used FIRST to segment subcortical regions in 79 individuals with manifest HD and 30 controls, and van den Bogaard et al. (2011) used FIRST in the TRACK‐HD sample comprising 30 individuals with premanifest HD, 30 with manifest HD, and 30 controls. In both studies, no group differences were found in FIRST‐segmented amygdala volumes, but smaller volumes were found in the HD groups compared with controls in several regions including nucleus accumbens, caudate, putamen, and hippocampus. For studies such as these where multiple brain regions are investigated in relatively large samples, manual segmentation may prove impracticably time consuming. However, it is possible that FreeSurfer may provide an automated option with the ability to produce amygdala segmentations more delineable between groups.

The lack of group differences in amygdala volumes derived from automated methods may be partially understood by exploring estimation biases for the automated methods. Bland–Altman plots indicated that all automated segmentation approaches produced a bias wherein the overestimation of volume was most severe for smaller amygdalae. This bias was least pronounced for FreeSurfer, strongest for FIRST, and somewhat reduced for ANTS/FIRST. A similar pattern of bias was found by Schoemaker et al. (2016) in their paediatric sample, where FIRST overestimated volumes of smaller structures more severely than FreeSurfer. Amygdalae in individuals with HD are smaller than amygdalae in controls as a result of atrophy. Accordingly, the upshot of this estimation bias was most apparent in the symp‐HD group, where the average differences between manual and FIRST‐derived volumes (as per Table 2) for this group were 96% (left amygdala) and 93% (right amygdala). By contrast for pre‐HD differences were 89% (left) and 88% (right), and for controls 61% (left) and 60% (right). This bias appears to be a major factor contributing to the inability to detect differences in volumes between groups for segmentations produced with FIRST.

In terms of the methodological mechanism of this bias, speculatively, it is possible that FIRST's model could not accurately conform amygdala meshes to amygdalae that were abnormally small due to atrophy. The Bayesian modeling approach employed in the default FIRST pipeline allows shape meshes of each structure to deform further than the boundaries of the structures in the training set, to fit the observed anatomy (Patenaude et al., 2011). Feng et al. (2017) suggested that, particularly in cases of brain abnormality, the use of linear rather than nonlinear registration in the initial steps of FIRST's pipeline could result in a structure in the model being inaccurately aligned with the same structure in the target data, in ways that the mesh deformation cannot fully correct for. Feng and colleagues improved segmentation accuracy with FIRST by incorporating initial nonlinear transformations and additional quantitative susceptibility mapping data. In this study, we performed initial ANTS nonlinear registration to the MNI template in an attempt to reduce the distance between the model and the underlying (albeit bias corrected, nonlinearly transformed, and resampled) anatomy. This step appears to have reduced some of the differences apparent between manually segmented volumes and those produced by FIRST, as can be seen in Figure 4. However, any improvement in mesh fitting conferred by this nonlinear registration did not prevent significant overall volume overestimation. The bias toward more severe overestimation of smaller amygdala also remained, but was somewhat reduced. This reduction in bias, in turn, may have led to slight improvement in ability of ANTS/FIRST to differentiate between groups. As mentioned by Perlaki et al. (2017), continued evaluation of FreeSurfer and FIRST in future will be useful, as they are actively developed. Amygdala‐specific segmentation techniques such as those by Collins and Pruessner (2010), Hanson et al. (2012), and Saygin et al. (2017) should also be evaluated in HD, and may provide promising alternatives in HD studies investigating amygdala structure.

FreeSurfer segmentations contained an additional bias where right amygdala segmentations were larger than left amygdala segmentations. This bias was seen in control participants as well as in the HD groups, indicating that this result was not indicative of lateralized atrophy. We did not find a lateralized volume bias for any of the other segmentation methods, including manual segmentation. This suggests that it is unlikely to be due to a genuine volume difference, which manual segmentation should have detected. With respect to lateralization in HD, although there are isolated reports of left lateralized atrophy in the striatum (Minkova et al., 2017; Mühlau et al., 2007) and cortex (Rosas et al. (2002), HD is not considered a lateralized disorder and there is no strong evidence for a hemispheric bias in HD neurodegeneration. It is also unlikely to be due to image artifact, unless a subtle artifact was present that solely affected segmentation by FreeSurfer. To the best of our knowledge, there is no published evidence for a right hemisphere volume bias produced by FreeSurfer in the amygdala specifically. However, Fennema‐Notestine et al. (2007) identified a right‐dominant asymmetry in hippocampal volumes in two‐thirds of FreeSurfer's manually traced atlas training set, and a concomitant right hemisphere volume bias when performing hippocampal labeling with FreeSurfer. Given that the hippocampus and amygdala are adjacent structures with similar tissue intensity values, it is possible that a similar asymmetry in amygdala volume could exist in FreeSurfer's training set due to anatomy or otherwise, which could be translated as a right volume bias when labeling new amygdalae. It may be useful to note the current finding in relation to future studies involving amygdala in HD with FreeSurfer segmentation. Clearly, a bias of this type would be problematic in any study investigating lateralization of amygdala volume or structure, or where the segmentation may be used as a mask to investigate amygdala connectivity or function where hemispheric differences are key features. However, where lateralization is not the main property of interest, a potential lateralized bias in volume could be accepted in view of other advantages that FreeSurfer may offer.

Another factor to consider in choosing the most appropriate segmentation method is sample size, which affects statistical power. For the current data, sample size estimation indicated that in order to reproduce the statistical differences that were observed in manually segmented amygdala volume between groups using automated methods, group sizes would need to be substantially larger. These differences were less pronounced for large effect sizes, such as the notable volume differences between symp‐HD and controls, which reflects the more advanced atrophy in symp‐HD. By contrast, the difference in sample size required to differentiate amygdala volumes between groups was particularly marked for comparisons between pre‐HD and symp‐HD. If *p* = .05 and power = 0.8 were assumed, sample sizes over 10 times larger would be required for automated methods. The substantially larger sample size required for comparison between different stages of HD reflects the subtle changes in amygdala atrophy that occur as the disease progresses from presymptomatic into symptomatic stage (Ahveninen et al., 2018).

Sample sizes required to delineate between controls and symp‐HD were smaller for ANTS/FIRST than for FreeSurfer. This appears unintuitive considering that FreeSurfer was able to statistically differentiate amygdala volumes between these groups more effectively than ANTS/FIRST. Interpretation of this discrepancy may be assisted by considering the variances of each subset of the data presented in Figure S1. This figure illustrates that automated methods incur greater variance and irregular distributions due to labeling errors, which may then affect statistical comparisons. We calculated sample sizes using parametric statistics. We had established that although the majority of subsets of volume measurements in the current data were normally distributed, one sixth were not. Therefore, sample sizes we provide here should be interpreted as approximate indications rather than prescriptive.

### 4.1 Summary and conclusions

The current study utilized a large and balanced sample of individuals with HD and controls, for which manual segmentation of amygdala was performed. Manual segmentation provided gold standard volumetric data against which to assess existing automated segmentation protocols, and one experimental method. We found that manual segmentation is the most optimal method of amygdala segmentation in HD, producing volumes that were most easily differentiated between groups. Manual segmentation may be necessary in studies aimed at detecting amygdala volume differences between individuals with pre‐HD and symp‐HD, though a slightly larger sample size may be needed. FreeSurfer performed better than other automated methods on some measures and may constitute a favorable automated alternative. However, the introduction of a potential hemispheric bias in volume estimation may be problematic in studies investigating lateralization of amygdala volume change in HD. FIRST produced volumes that were closer in absolute volume to manual segmentations, but more strongly overestimated the volume of smaller amygdalae, and performed poorly in terms of differentiating amygdala volume between groups. Performing initial ANTS nonlinear registration with FIRST only somewhat improved accuracy compared to FIRST alone. When choosing segmentation methods for the amygdala in HD, options should be considered in context of the aim of the analysis, and the sample size available. The current data provide information to this end, and may also be informative in interpreting existing volumetric findings regarding the amygdala in HD.

## Supporting information


**Data S1**: Supporting Information.

## Data Availability

The derived data that support the findings of this study are available on request from the corresponding author. The MRI data are not publicly available due to ethical restrictions.
