# Supplementary material for: Accuracy of automated amygdala MRI segmentation approaches in Huntington's disease in the IMAGE‐HD cohort
Source: Hum Brain Mapp. 2020 Feb 7;41(7):1875–88. doi: 10.1002/hbm.24918 (PMC7268083; doi:10.1002/hbm.24918)
Supplement: Supplementary file 1 — Data S1: Supporting Information. [file HBM-41-1875-s001.docx]

**Supplementary material**

Amygdala segmentation protocol descriptions from Velakoulis et al. (2006) and Filipek et al. (1994)

Filipek et al. specify that the amygdala is “defined superiorly by the interface with the ansa peduncularis, medially by the CSF interface, inferolaterally by white matter, and inferomedially by a secondary border connecting the medial tip of the subjacent white matter with the uncal notch…. the secondary ventromedial transition from the amygdala anteriorly to the hippocampus posteriorly… was subsequently calculated as occurring at 10/24 of the distance between the AC and PC for each brain” (p. 346).

Velakoulis et al. specify, “The amygdala boundaries were as follows: posterior, appearance of amygdala gray matter above the temporal horn; superior-lateral, the thin strip of white matter that separates the amygdala from the claustrum and the tail of the caudate; medial, the angular bundle, which separates the amygdala from the entorhinal cortex; superior-medial, the semilunar gyrus; inferior, the hippocampus; inferior-lateral, the temporal lobe white matter and the extension of the temporal horn; and anterior, the section anterior to the appearance of the optic chiasm” (p. 142).

Fig_S1_here

*Supplementary Figure S1. Density plots of amygdala volume for each segmentation type, shown separately for right and left amygdalae, and broken down into groups. Density plots are smoothed histograms that assist in visualising the shape of distributions. Here it may be noted from visual inspection that for some subsets of the data, amygdala volumes are not normally distributed, as discussed further in section 2.4.1 and section 4 of the main text.*

Fig_S2_here

Supplementary Figure S2. Sample size estimation for ability to statistically detect amygdala volume differences between groups. Based on observed power and observed p-values (left), and power = 0.8 and p = .05 (right) for comparisons between a) control and pre-HD, b) control and symp-HD, and c) pre-HD and symp-HD. Estimates assume parametric data, and are based on t-tests performed on right hemisphere amygdala volumes. Vertical black lines indicate observed mean amygdala volume difference between groups in mm^3^.

Supplementary Table 1. Group comparisons of amygdala volumes for each segmentation method

| Segmentation type | L/R | Group comparison | *W* | *p* (uncorr) | Estimate | Lower CI | Upper CI | *p* (FDR) | *p* (Bonferroni*)* |
| --- | --- | --- | --- | --- | --- | --- | --- | --- | --- |
| Manual seg. | Left | **con vs. pre-HD** | **913.5** | **0.000** | **196.12** | **101.23** | **281.28** | **0.001** | **0.003** |
|  |  | **con vs. symp-HD** | **1078.5** | **0.000** | **264.94** | **184.53** | **344.71** | **0.000** | **0.000** |
|  |  | pre-HD vs. symp-HD | 789 | 0.068 | 77.85 | -9.61 | 147.37 | 0.136 | 1.000 |
|  | Right | **con vs. pre-HD** | **880** | **0.001** | **162.86** | **73.04** | **265.90** | **0.003** | **0.015** |
|  |  | **con vs. symp-HD** | **1026.5** | **0.000** | **236.43** | **160.82** | **333.82** | **0.000** | **0.000** |
|  |  | **pre-HD vs. symp-HD** | **819** | **0.030** | 84.90 | 8.33 | 155.05 | 0.079 | 0.709 |
| FreeSurfer | Left | **con vs. pre-HD** | **802.5** | **0.013** | **134.07** | **31.40** | **238.99** | **0.039** | 0.311 |
|  |  | **con vs. symp-HD** | **931** | **0.000** | **253.73** | **135.19** | **358.80** | **0.001** | **0.003** |
|  |  | pre-HD vs. symp-HD | 784.5 | 0.077 | 111.90 | -8.33 | 233.86 | 0.141 | 1.000 |
|  | Right | **con vs. pre-HD** | **773** | **0.033** | **146.73** | **11.53** | **273.59** | 0.080 | 0.795 |
|  |  | **con vs. symp-HD** | **854** | **0.005** | **170.65** | **62.79** | **324.21** | **0.019** | 0.111 |
|  |  | pre-HD vs. symp-HD | 713 | 0.346 | 52.54 | -58.95 | 155.70 | 0.437 | 1.000 |
| FIRST | Left | con vs. pre-HD | 690 | 0.257 | 61.51 | -55.74 | 171.07 | 0.342 | 1.000 |
|  |  | con vs. symp-HD | 729 | 0.171 | 85.22 | -45.49 | 204.39 | 0.274 | 1.000 |
|  |  | pre-HD vs. symp-HD | 668 | 0.666 | 18.22 | -102.52 | 144.80 | 0.713 | 1.000 |
|  | Right | con vs. pre-HD | 551 | 0.602 | -46.33 | -192.22 | 101.88 | 0.688 | 1.000 |
|  |  | con vs. symp-HD | 546 | 0.438 | -60.02 | -198.62 | 81.37 | 0.526 | 1.000 |
|  |  | pre-HD vs. symp-HD | 614 | 0.859 | -7.69 | -143.52 | 110.85 | 0.859 | 1.000 |
| ANTS/FIRST | Left | con vs. pre-HD | 696 | 0.230 | 58.95 | -39.72 | 162.10 | 0.342 | 1.000 |
|  |  | **con vs. symp-HD** | **843** | **0.007** | **157.62** | **53.18** | **262.70** | **0.023** | 0.162 |
|  |  | pre-HD vs. symp-HD | 774 | 0.099 | 99.14 | -20.50 | 205.03 | 0.169 | 1.000 |
|  | Right | con vs. pre-HD | 630 | 0.683 | 31.02 | -110.84 | 164.67 | 0.713 | 1.000 |
|  |  | con vs. symp-HD | 776 | 0.055 | 112.33 | -2.56 | 223.61 | 0.119 | 1.000 |
|  |  | pre-HD vs. symp-HD | 731 | 0.250 | 74.03 | -55.10 | 201.83 | 0.342 | 1.000 |

Note: *W* is the test statistic for the Wilcoxon rank sum test. ‘Estimate’ is the median difference between a sample from each compared group. ‘Con’ = control, ‘pre-HD’ = presymptomatic HD, ‘symp-HD’ = symptomatic HD. Lower and upper CI: 95% confidence intervals of the estimate. ‘Uncorr’= uncorrected, ‘FDR’ = False Discovery Rate corrected.

Supplementary Table 2. Comparisons of right versus left amygdala volumes in each segmentation method

|  |  | *V* | *p* (uncorr.) | *p* (FDR) | Pseudo-  median | Lower CI | Upper CI | *r* | *p* (Bonf.) |
| --- | --- | --- | --- | --- | --- | --- | --- | --- | --- |
| Manual seg. | All groups | 2556.5 | 0.471 | 0.831 | -8.97 | -32.36 | 15.38 | -0.005 | 1.000 |
|  | Control | 306.0 | 0.893 | 0.893 | 4.48 | -55.10 | 56.38 | 0.086 | 1.000 |
|  | Pre-HD | 275.0 | 0.522 | 0.831 | -11.53 | -50.30 | 25.31 | 0.004 | 1.000 |
|  | Symp-HD | 298.0 | 0.592 | 0.831 | -9.45 | -43.57 | 24.99 | 0.016 | 1.000 |
| FreeSurfer | **All groups** | **598.0** | **0.000** | **0.000** | **-122.06** | **-148.33** | **-94.83** | **-0.475** | **0.000** |
|  | **Control** | **111.0** | **0.001** | **0.004** | **-97.07** | **-164.03** | **-41.65** | **-0.214** | **0.016** |
|  | **Pre-HD** | **72.0** | **0.000** | **0.000** | **-105.24** | **-147.05** | **-60.87** | **-0.284** | **0.000** |
|  | **Symp-HD** | **33.0** | **0.000** | **0.000** | **-154.41** | **-191.58** | **-120.78** | **-0.360** | **0.000** |
| FIRST | All groups | 2250.5 | 0.089 | 0.204 | -37.80 | -81.05 | 6.41 | -0.093 | 1.000 |
|  | Control | 340.0 | 0.478 | 0.831 | 36.84 | -67.60 | 142.56 | -0.004 | 1.000 |
|  | Pre-HD | 206.0 | 0.075 | 0.201 | -49.34 | -113.41 | 6.41 | -0.099 | 1.000 |
|  | **Symp-HD** | **187.5** | **0.023** | 0.073 | **-73.37** | **-152.49** | **-9.93** | **-0.138** | 0.364 |
| ANTS/FIRST | All groups | 2871.0 | 0.779 | 0.831 | 4.16 | -30.43 | 39.72 | 0.053 | 1.000 |
|  | Control | 325.0 | 0.648 | 0.831 | 14.74 | -45.17 | 81.05 | 0.026 | 1.000 |
|  | Pre-HD | 290.0 | 0.692 | 0.831 | -9.61 | -66.64 | 43.89 | 0.035 | 1.000 |
|  | Symp-HD | 353.0 | 0.762 | 0.831 | 11.53 | -57.02 | 77.53 | 0.049 | 1.000 |

Note: results are for Wilcoxon signed-rank tests. ‘V’ is the test statistic. This test is described further in section 2.4 of the main text.
